# A critical evaluation of the index of patch quality

**DOI:** 10.1098/rspb.2021.0459

**Published:** 2021-05-26

**Authors:** Annette L. Fayet, Alasdair I. Houston

**Affiliations:** ^1^ Department of Zoology, University of Oxford, Mansfield Road, Oxford OX1 3SZ, UK; ^2^ School of Biological Sciences, University of Bristol, Life Sciences Building, 24 Tyndall Avenue, Bristol BS8 1TQ, UK

**Keywords:** diving, index of patch quality, inverse optimality, gain function

## Abstract

The inverse optimality approach can allow us to learn about an animal's environment by assuming their behaviour is optimal. This approach has been applied to animals diving underwater for food to produce the index of patch quality (IPQ), which aims to provide a proxy for prey abundance or quality in a foraging patch based on the animal's diving behaviour. The IPQ has been used in several empirical studies but has never been evaluated theoretically. Here, we discuss the strengths and weaknesses of the IPQ approach from a theoretical angle and review the empirical evidence supporting its use. We highlight several potential issues, in particular with the gain function—the function describing the energetic gain of an animal during a dive—used to calculate the IPQ. We investigate an alternative gain function which is appropriate in some cases, provide a new model based on this function, and discuss differences between the IPQ model and ours. We also find that there is little supporting empirical evidence justifying the general use of the IPQ and suggest future empirical validation methods which could help strengthen the case for the IPQ. Our findings have implications for the field of diving ecology and habitat assessment.

## 1. Introduction

Air-breathing animals that hunt for food underwater and return to the surface for air are referred to as divers. Optimal diving theory attempts to predict their behaviour, given the possible foraging options and the constraint imposed by a limited ability to store oxygen. Thus, the approach uses the environmental parameters to predict behaviour. Mori *et al*. [1] reverse this direction of analysis; starting from the assumption that behaviour is optimal, they find the value of an environmental foraging parameter that predicts such behaviour. This is an example of inverse (or reverse) optimality [2–5]. The authors take the parameter to be an index of patch quality (hereafter IPQ). In this paper, we provide a critical evaluation of the IPQ, including specific issues with the IPQ model, with its empirical validation, and also surrounding the use of the inverse optimality principle in this context of diving animals.

## 2. The index of patch quality

Optimal diving models can be divided in two types [6]. Models of one type are based on items; these are appropriate for divers that make decisions about whether to accept or reject items that they encounter during a dive. Several models consider divers that return to the surface after capturing an item, known as single-prey loaders [7–10], as opposed to divers that return to the surface with several items, which are multiple-prey loaders. These models predict whether an item should be accepted as a function of the duration of a dive, and when an unsuccessful dive should be abandoned. The second type of optimal diving models is based on time allocation. These models assume that the gain from a dive is a function of time spent foraging and predict the optimal value of this time [11–14].

The IPQ is derived from the second type of models. At the heart of these models is the performance over a dive cycle. A dive cycle comprises a dive of duration *u* followed by a pause at the surface of duration *s*; *u* is equal to *t* *+* *τ,* where *t* is the time spent foraging under water and *τ* the time travelling to/from the foraging depth at the start and end of the dive; as such *τ* is usually determined by depth [1,15]. The surface pause duration aims to replenish the oxygen reserves depleted by the dive and is, therefore, a function of *t* and *τ* [12]; for simplicity, we assume that *s* depends on *t* + *τ* = *u* as in other studies (e.g. [1,15]). A key aspect of these IPQ models is that gain (energy intake) depends on the time spent foraging *t*, which is reflected in a gain function *g*(*t*) representing the energy gain from a particular dive. If all patches have the same gain function, then the diver's gross rate of energy gain *R*(*t*) is given by the equation

2.1 $$R(t)=\;\frac{g(t)}{t+\;\tau +s(t+\tau )}$$

and is maximized at t*. Inverse optimality assumes that the observed foraging time is the optimal foraging time t*. From equation (2.1), it follows that this time is given by

2.2 $$(t^{*}+\;\tau +\;s)\;g^{\prime }=g(1+s^{\prime }),$$

where all functions are evaluated at t*. In the most basic case, gain is proportional to *t*, i.e. *g*(*t*) = *Bt*, where *B* is the rate of gain. In this case, *R* is maximized by maximizing the proportion *P* of the time spent actively foraging during the dive cycle [11,12]. The consequences of maximizing

$$P=\frac{t}{\;(t+\;\tau +s)}$$

are presented in [11], which also considers a gain function that is instead a power function of the time spent foraging:

2.3 $$g(t)=At^{x},$$

where *A* and *x* are positive constants. The parameter *x* represents how the returns experienced by an animal change throughout the duration of a foraging dive, while *A* scales this time-dependent part of *g* and is likely to reflect the abundance of prey, their size, and energy content. The effect of *x* can be understood by noting that

2.4 $$g^{\prime }(t)=xAt^{x-1}$$

and

2.5 $$g^{\prime \prime }(t)=(x-1)xAt^{x-2}.$$

From equation (2.4), *g*(*t*) is increasing and from equation (2.5), it is accelerating if *x* > 1 and decelerating if *x* < 1. Thus, *x* characterizes the increasing or diminishing returns an animal experiences as it continues to feed, perhaps caused by local changes in prey density such as prey aggregating, moving away, or being captured [16,17]. Note that *x* is a number—it has no dimensions. It is this exponent *x* which is defined to be the IPQ (others have used ln(*x*), e.g. [15]). Subsequent studies have used this function to determine patch quality from animal diving data collected with depth loggers [16,18–20].

As pointed out in [1], *A* has no effect on t*. If behaviour is optimal, i.e. equation (2.2) holds, and gain is given by equation (2.3), we obtain

2.6 $$x=\frac{t^{*}(1+s^{\prime })}{\;(t^{*}+\tau +s)}$$

where *s* and *s*′ are evaluated at t*. Given *s*, we can then calculate *x* from equation (2.6). This procedure can be followed for any *t*, but it assumes a model based on time allocation, i.e. the time foraging determines the gain, so it is not appropriate if the dive involves catching a single item, and may, therefore, not be suitable for studies of single-prey loading species.

Calculating *x* requires describing the surface pause duration *s* as a function of *u.* Two functions have been commonly used. One is

2.7 s(u)=bexp(cu),

from [15,20], where *b* and *c* are fitted constants (note the surface time is not 0 at *u* = 0). A simple version of the other follows from

2.8 u=K(1−exp⁡(−αs)),

where *K* characterizes the maximum oxygen intake and is used in multiple studies [1,21] (for a version with the metabolic rates during travel time and foraging time see [11]). From equation (2.8), we get

2.9 $$s(u)=\frac{1}{\alpha }\;\ln\left[\frac{K}{K-u}\right]\;.$$

**Figure 1.**
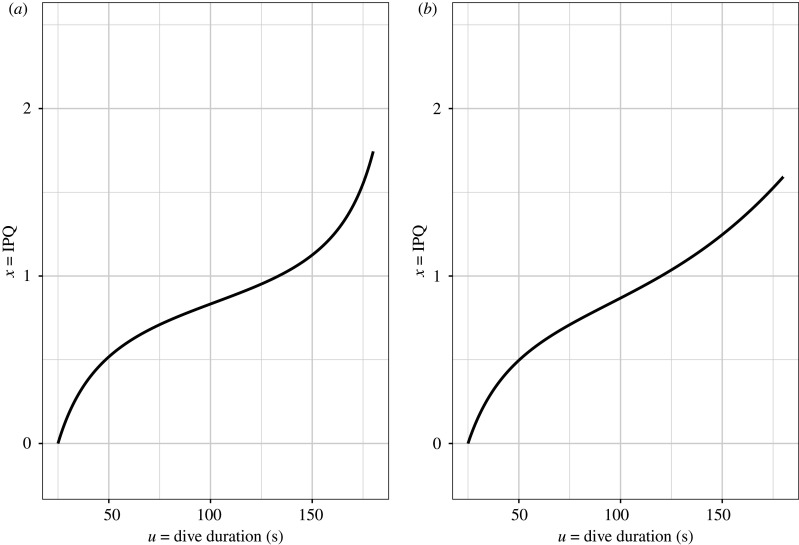
IPQ as a function of *u* for a travelling time *τ* = 25 s and the surface time function *s*(*u*) in equation (2.7) (*a*) and equation (2.9) (*b*). Parameter values are taken from [15] (*b* = 3.18, *c* = 0.019, *a*) and [1] (*K* = 202, *α* = 0.02, *b*).

The equation for *x* when *s* is given by equation (2.7) or (2.9) can be found in the electronic supplementary material, appendix S1. In both cases, *x* follows a logit-like curve, increasing steeply with dive duration both early and late in a dive (figure 1).

Generally, and as shown by this description of the IPQ model as a particular case, the inverse optimality framework to modelling the behaviour of animals diving for food involves an assumption about the gain function *g*(*t*), the surface pause duration *s*(*u*), and a currency (typically gross rate *R* in the IPQ model). An index of quality (*x* in the IPQ model) is then calculated from the observed time foraging. The use of inverse optimality has a key benefit: it provides an opportunity to learn about an animal's environment simply by observing its behaviour. As explained in [22], ‘If animals behave optimally towards a given environment and we know the relationship between the optimal behaviours in a set of environmental conditions, then we can estimate the environmental conditions by observing the behaviours of the animals’*.* In the case of the IPQ, an objective of the measure is to allow the estimation of prey abundance at a foraging patch from dive data, without requiring actual measurements of prey abundance. This is particularly useful because measuring prey abundance *in situ* is costly and difficult, and environmental variables available from satellite data, such as chlorophyll A or net primary productivity, are not always valid proxies of prey abundance [23]. The alternative method of using animal-borne cameras to measure prey abundance is currently limited to large diving animals, so is inapplicable to most diving species like seabirds. In theory, the IPQ could, therefore, be a very useful tool to study the ecology of diving animals.

## 3. Potential issues with the index of patch quality

However, the use of the IPQ as a proxy for environmental quality raises some questions. First, from equation (2.6), we can see that the IPQ is independent of *A*, despite the fact that *A* might depend on prey abundance. This is an issue because while the optimal foraging time t* is independent of *A*, the resulting optimal rate *R*(t*) does depend on *A*. This issue is not limited to the IPQ model, but applies whenever all patches are the same [24], their gain function is the product of *A* and a function of *t*, and the currency is *R*. When the IPQ was first described in [1], the authors used a figure representing *g*(*t*) in which *A* was the same for all curves. However, if *A* varies with *x*, then a low value of *x* alone does not indicate poor quality, because *A* could be high. This point holds if *A* is interpreted as prey abundance or energy content.

In fact, from equations (2.3) and (2.4), we obtain

3.1 $$x=\;\frac{g^{\prime }(t)}{g(t)/t}$$

with *g*(*t*)/*t* representing the average gain rate. As such, *x* represents the instantaneous gain rate divided by the average gain rate. We return to this average rate of gain later as a way to characterize foraging opportunity.

Another problem relates to unclear definitions of patch quality itself. The original study describing the IPQ does not define patch quality [1], and one cited reference interprets quality as prey density [14], which differs from *x* and may depend on *A*. This shows that patch quality is not an intuitive parameter to define [24] and highlights the need for studies using the IPQ to clearly define quality and keep this definition in mind when interpreting results.

A third issue arises from the fact that diving parameters, and in particular the time allocation from which the IPQ is derived, may be influenced by other factors, so that the IPQ may not only reflect local prey conditions, but how the diver uses those prey conditions. This is supported by findings showing differences in IPQ between male and female common guillemots *Uria aalge* during the male-only parental care period, but no difference outside that period [25,26]. Here, differences in IPQ may reflect the fact that the males are under different pressures from females because of chick-rearing duties. Consequently, (i) the IPQ may only allow meaningful comparisons of patch quality for individuals under similar conditions (e.g. same sex, breeding stage, etc.), a point noted in [21] but which seems to have been mostly ignored since, and (ii) caution is required when interpreting the IPQ as purely reflecting patch quality.

Finally, and importantly, the assumption that the gain rate follows a power function may not always be correct. Although equation (2.3) was fitted to data from bouts of diving by elephant seals *Mirounga angustirostris* [16], using vertical excursions as indications of encounters with prey, data from Adelie penguins *Pygoscelis adelia* suggest that equation (2.3) may not be adequate. Miniature cameras fitted on foraging penguins showed sigmoid gain functions in 55% of all dives (versus 18.5% for purely accelerating or decelerating functions) [17], in other words, the gain function first accelerates as time spent foraging increases but then decelerates. Sigmoid gain functions were also observed in razorbills *Alca torda* and common guillemots [27]. A sigmoidal gain function could be represented by

3.2 $$g(t)=\;\frac{At^{n}}{T^{n}+\;t^{n}}\;.$$

**Figure 2.**
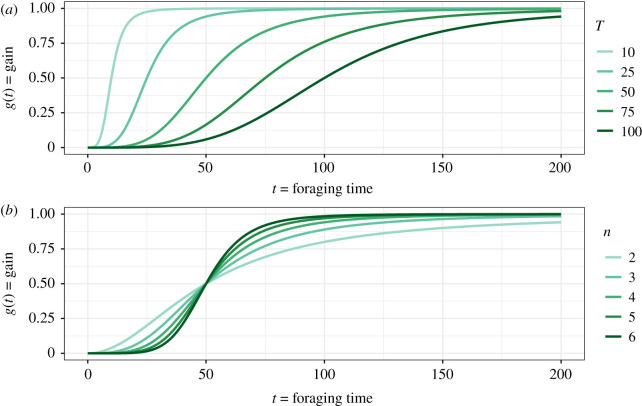
(*a*) Gain function *g*(*t*) as a function of foraging time *t* calculated from equation (3.2), for different values of *T* (represented by different colours), *n* and *A* fixed at *n* = 4 and *A* = 1. (*b*) Gain function *g*(*t*) as a function of foraging time *t* calculated from equation (2.9), for different values of *n* and for *T* fixed at *T* = 50 and *A* = 1. (Online version in colour.)

**Figure 3.**
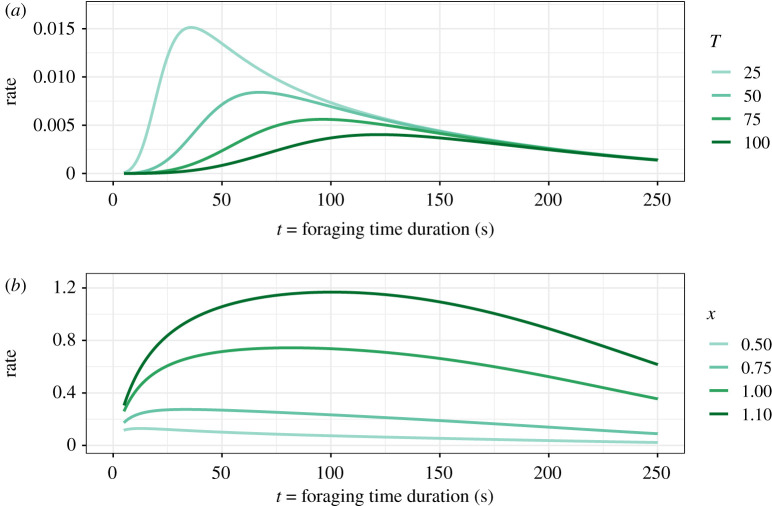
(*a*) Gross rate of energy gain *R* as a function of foraging time duration *t*, calculated from equations (2.1), (3.2), and (2.7), for different values of *T*, *n* = 4, *τ* = 10 s, and other parameters as in figure 1. (*b*) Gross rate of energy gain *R* as a function of foraging time duration *t*, calculated from equations (2.1), (2.3), and (2.7), for different values of *x*, *n* = 4, *τ* = 10 s, and other parameters as in figure 1. The equivalent figures for *s* described by equation (2.9) can be found in the electronic supplementary material, figure S2. (Online version in colour.)

Examples of how gain *g*(*t*) varies with *t*, *T*, and *n* are shown in figure 2. As *t* increases from zero, *g*(*t*) increases towards *A*, passing through *A*/2 at *t* = *T*. An increase in *T* decreases gain and has the general effect of delaying its steep rise (figure 2*a*, in which *n* is fixed). Increasing *T* decreases gain, in other words, ∂g/∂T<0, and therefore, 1/*T* is a metric of quality, as defined in [24]. *n* characterizes how step-like the gain function is, i.e. how steeply foraging starts to pay off as foraging time increases. The greater the value of *n*, the more step-like the gain function (figure 2*b*, in which *T* is fixed). The figure shows that gain decreases with *n* for *t* < *T* but increases for *t* > *T*. For *n* fixed, the gross rate of energy gain *R* decreases with *T* (figure 3*a*). This figure also shows that t* decreases when quality (1/*T*) increases, unlike in the IPQ model (figure 3*b*).

**Figure 4.**
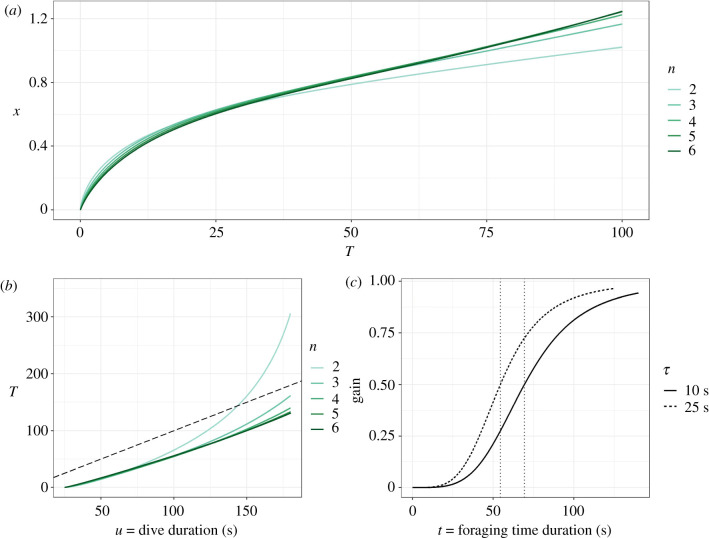
(*a*) The IPQ *x* as a function of *T* calculated from equations (2.6), (2.7), and from electronic supplementary material, appendix S2, for different values of *n*. Other parameters as in figure 1. (*b*) *T* as a function of dive duration *u* calculated from equations (2.6), (2.7), and (3.2), for different values of *n*. The dashed line represents *T* = *u*. (*c*) Gain function *g*(*t*) as a function of time foraging *t* calculated from equation (3.2), and for *s* described by equation (2.7), for *n* = 4, *A* = 1, *u* = 100 s, and for two values of *τ*. Other parameters as in figure 1. The vertical dotted lines represent *t* = *T* for each *τ*. (Online version in colour.)

To find an IPQ, we go from *t* to a parameter of the gain function. When *g* is given by equation (2.3), *t* uniquely determines *x*, independently of *A*. If *g* is given by equation (3.2), once again *A* has no effect, but there are two parameters (*n* and *T*) which do have an effect, so *t* does not determine a unique index. To get round this problem, we fix *n* and take *T* to be our index. By using equation (2.7) or (2.9) to describe *s*, we can use equations (2.6) and (3.2) to derive *T* as a function of dive duration (electronic supplementary material, appendix S2). Some examples are given in figure 4*b* which shows the effect of *n* on *T* decreases as *n* increases. Because the animal may not stay under water for a very long time, it is important to establish what part of the sigmoidal curve *g*(*t*) would be observed. From figure 4*b*, we see that unless *n* is small, the dive duration *u* exceeds *T*, and hence some deceleration of gain is likely to be observed unless *τ* is large. This is illustrated in figure 4*c*, which describes *g*(*t*) up until the value of *t* at which it is optimal for the animal to end the dive and return to the surface. This figure shows how, for a given dive duration and *n*, *g*(*t*) depends on the duration of the dive, and indeed follows a sigmoidal curve. Because *u* is fixed, if *τ* increases, the animal can spend less time foraging, so *T* is reached sooner and the deceleration of gain occurs earlier. Similar plots for *s* described by equation (2.9) are shown in the electronic supplementary material, figure S1.

## 4. Empirical validations of the index of patch quality

An index of the quality of a patch should reflect energy gain, and how good a feeding area is in terms of prey abundance and/or value (e.g. prey size or energy density). In the gain function given by equation (2.3), these factors could be captured by *A*, but *A* is not included in the formula of IPQ (equation (2.6)). Several studies have calculated the IPQ and some have attempted to validate it as a good proxy for patch quality in diving birds and mammals (table 1). These studies used different methods, but generally measured other variables potentially indicative of prey abundance or quality and calculated their correlation with the IPQ. Direct measures of prey abundance at the time and location of the dive are difficult to collect simultaneously with diving behaviour. To our knowledge, a single study so far has attempted this, by simultaneously deploying depth loggers with animal-borne cameras in seals [21]. Another study, also on seals, combined diving data with prey abundance data from a ship-based survey [19], while a study of diving seabirds estimated prey mass from the observation of birds upon their return from a foraging trip [15]. All other studies measured correlations between IPQ and indirect measures such as number of dives in a bout.

**Table 1.** List of studies calculating the IPQ in diving animals and their findings regarding the relationship between the IPQ and direct or indirect measures of prey abundance. n.s.: non significant; p.e.: parameter estimate.

species	IPQ (range)	context	correlation	study
Brünnich's guillemot *Uria lomvia*	*x* ∈ [0.1, 1.6]	chick-rearing	IPQ at a given depth increases with the frequency of dives per bout at that depth (Kendall's *τ* = 0.31, *p* < 0.05)	Mori *et al*. [1]
	ln(*x*) ∈ [0.08, 1.65]	chick-rearing	IPQ increases with prey mass caught on the last dive (slope = 0.18 with ln(prey mass), *R*^2^ = 0.161)	Elliott *et al*. [15,28]
	ln(*x*) ∈[−0.1, −1.2]	post-fledging chick care	IPQ decreases over time from −0.8 to −1.2 over ∼6 weeks	Elliott & Gaston, [18]
common guillemot *Uria aalge*	ln(*x*) ∈[0.2, 0.6]	chick-rearing	n/a (IPQ of females > IPQ of males)	Burke *et al*. [26]
	ln(*x*) ∈[−1, −0.2]	chick-rearing	n/a (IPQ of females > IPQ of males)	Elliott *et al*. [25]
Razorbill *Alca torda*	ln(*x*) ∈ [0.12, 0.16]	incubation and chick-rearing	IPQ independent of the frequency of dives in bout but decreased with dive-pause ratio of dive bouts (p.e.: −0.04 ± 0.01, ΔAIC = −10) IPQ increases with distance from the colony (p.e.: 24.76 ± 23.74, ΔAIC = −12)	Shoji *et al*. [20]
black guillemot *Cepphus grylle*	ln(*x*) ∈ [0, 0.68]	chick-rearing	frequency of dives in bout independent of IPQ	Shoji *et al*. [29]
Antarctic fur seal *Arctocephalus gazella*	*x* ∈ [0.24, 0.45]	breeding females	IPQ increases with krill abundance measured from survey (rank correlation = 1.0, *p* = 0.045)	Mori & Boyd, [19]
Weddell seal *Leptonychotes weddellii*	*x* ∈ [0, 1.4]	breeding females	IPQ increases with prey abundance measured from camera (Max prey index: Spearman's rank correlation *r_s_* = 0.68, *p* < 0.001 site 1, *r_s_* = 0.42, *p* < 0.05 site 2. Mean prey index: *r_s_* = 0.64, *p* < 0.01, n.s. site 2)	Mori *et al*. [21]

Most studies found positive correlations between the IPQ and other measures, of varying strengths (table 1). The strongest validation of the IPQ so far comes from a positive correlation between IPQ and maximum prey abundance as measured from on-board cameras [21]. Most other studies found at least one positive correlation between IPQ and a proxy for prey abundance, but also lack of correlations with other variables. For example, a study found the IPQ was positively correlated with the mean prey abundance at only one of two study sites [21], while others found no evidence that the IPQ correlated with the number of dives per bout [20,29]. Although the majority of these studies found positive correlations between the IPQ and other potential proxies of quality, the scarcity of validations from direct measures of prey abundance and the lack of a standard ‘behavioural’ variable correlated with across studies and species indicates results may still need to be interpreted with caution. For example, the number of dives per bout was used in three studies, but only correlated with IPQ in one of them (table 1).

Another issue with some of these validations is that they are performed on species which only bring a single prey item back to the nest, so the validation variable (e.g. prey mass) will only be based on one prey item. The IPQ is based on time allocation models, so is applicable to species which do not base their diving behaviour on what prey they find (e.g. its size), but instead have a gain function that relates to time and so the *g*(*t*) approximation used to calculate the IPQ fits nicely. At the other end of the prey-loading spectrum, single-prey loading species may base their diving behaviour on the prey they encounter and it may not be possible to relate their gain function to time spent on the dive. For example, they could be ‘picky’ and reject small prey items at the beginning of their dive, and settle for anything they find towards the end of the dive [8,9]. Other species, like razorbills, may sit in the middle of these two extremes and be facultative multiple-prey loaders [30]. They usually catch multiple items but if they find a large prey item they may collect it and end the dive. IPQ calculations are suitable for species on the high end of the prey-loading spectrum but become less suitable as species move towards the lower single-prey end of the spectrum (for work on indicators of quality in single-prey loaders, see [8,31]).

Attempts at validating the IPQ by testing for correlations between *x* and abundance assumes that abundance is the ‘real’ dimension of quality, and, therefore, that a good IPQ should correlate with it. However, patch quality can vary along several dimensions, so if *x* really is a relevant dimension, then it does not necessarily need to correlate with abundance. Instead, in order to provide convincing evidence that the IPQ does indeed characterize patch quality, the measure should be validated empirically by measuring an approximate *g*(*t*) to describe how gain increases over time as the dive goes on. This could be achieved by combining the dive data with *in situ* prey sampling or, even better, on-board cameras. Unfortunately, few studies using the IPQ also collected this sort of data (table 1), and none provided a gain curve. In fact, very few studies of animal diving behaviour seem to measure the gain function (e.g. [17,27]). In the absence of *in situ* prey sampling or on-board cameras, the next best option to validate the IPQ empirically would be to calculate the average gain rate *g*(*t*)/*t*, which reflects the energy gained on a dive and the time spent foraging on that dive and is proportional to *A* for a fixed *t*. Although this does not allow us to estimate *A* alone, because we do not know exactly how foraging depends on *t*, it would provide a measure that depends on *A*. In many species, seabirds in particular, this could be done by measuring—alongside diving behaviour to measure *t*—the load brought back to the offspring from a foraging trip, which should reflect the energy gained on the last dive or dive bout. This method was used in a single-prey loading species [15] but not, to our knowledge, in more appropriate multiple-prey loading species. Information about prey load can be collected by observing the nests of tagged birds during feeding watches [15]. Alternative methods include using motion-activated camera-traps near nests [32], manually weighing chicks after provisioning, or using automated weighing devices deployed on or near nests [33,34]. When such options are unavailable, it may be possible to estimate prey load from stomach probes [35], from fine-scale measurements of underwater movements during the bottom feeding time, such as ‘wiggles’ in penguins [36], or even measuring additional weight of the load from accelerometery data, which can sometimes be collected alongside dive data [37].

## 5. Discussion

Alongside potential issues with the IPQ models we have raised so far, a separate reservation relates to the use of inverse optimality in this context of diving animals. An underlying assumption of the IPQ approach is inverse optimality, i.e. the assumption that an animal's behaviour is always optimal even on a fine timescale. In our context of animals diving to catch prey, this principle assumes that the time spent foraging *t* on each individual dive is the optimal foraging duration under those circumstances (i.e. *t* = t*). Taken together with the IPQ approach [1], this is equivalent to saying that the animal has estimated *x* exactly and chosen the optimal foraging time for that value of *x*, and the IPQ procedure does not involve any independent check that this is the case. However, this assumption could fail for multiple reasons [21]. First, even if the gain function is well characterized by equation (2.3), stochastic events could disrupt the dive, e.g. a predator approaching, a collision with a conspecific aiming for the same prey item, or oxygen being consumed slightly faster than expected. Assuming every single dive is optimal is, therefore, a very strong, and possibly not very realistic, assumption. We note in this context that the rate curve for lower values of *x* is quite flat (figure 3*b*), suggesting that departures from the optimum are not very costly. Second, t* is only independent of *A* when maximizing the gross rate of gain, in a habitat where all patches are the same [24]. If the net rate of gain is considered, *A* has a slight effect on t* [11]. Unfortunately, working with net rate requires energy expenditure to be estimated, and unless *x* is known a given foraging time only identifies possible combinations of *A* and *x*. Together, these points raise questions about the applicability of the principle of inverse optimality underpinning the IPQ approach and show that this assumption should be made with caution.

Another point worth mentioning concerns the optimization criterion that we assume is being maximized. Instead of gross energy intake rate (energy gained divided by the time on the dive), this could be net energy intake rate (net energy gained divided by the time on the dive), energetic efficiency (the energy gained divided by the energy expended on the dive) [11], or a currency that depends on both energy and predation [38]. If, as in most of the literature we have cited, the rate of energy expenditure is assumed constant throughout the dive cycle, then efficiency becomes equivalent to intake rate so this does not matter. However, if different metabolic rates are used for the travelling and foraging phases of the dive (e.g. as in [11]) then which criterion the animal is assumed to maximize will affect optimal dive time and hence the IPQ.

In this paper, we simply use a gain function which allows for both increasing (*g* accelerating) and decreasing (*g* decelerating) returns over time, which has robust empirical support [17,27]. We emphasize that we do not claim that a sigmoid function should always be used as the gain function nor always gives better estimates than the IPQ model, we just provide an alternative which we believe is more appropriate in some cases. It is important to note that the issue we raised earlier about the independence between *A* and the IPQ still applies to this alternative model, as the gain function (equation (3.2)) remains the product of *A* and a function of time. Whether only the acceleration part of the curve is observed, or whether the animal stays underwater long enough to experience the decelerating gain, is an important question and depends on the two parameters *T* and *n*. We show that in most cases, the animal's optimal behaviour will result in both being observed (figure 4*c*). It may also be useful to note that the gain function we propose is unlikely to be accurate—for example, its symmetry may not occur in reality—nonetheless, this suffices to illustrate the issues that arise when the gain curve is not as simple as that given by equation (2.3).

This leads to a key difference between the two approaches. The indices from both the IPQ approach (*x*) and ours (*T*) increase with *t* (figures 1 and 3); in fact, *x* and *T* are correlated and increase together (figure 4*a*, see equation in the electronic supplementary material, appendix S2). However, their relationships with the gain function are opposite: while *g*(*t*) increases with *x* in the IPQ approach for *t* > 1 (equation (2.3)), in ours, it decreases with *T* (equation (3.2)). In other words, while a larger value of *x* represents better foraging conditions, greater values of *T* represent worsening conditions; this is also illustrated in figure 3*a*. To avoid confusion, it is important to note that the correlation between *x* and *T* illustrated in figure 4*a* only represents the mathematical relationship between the two indices and not an empirical situation, since each index is based on a different gain function, only one of which (at most) can be correct in any given situation. Figure 3*a* also illustrates how t* increases as quality (1/*T*) decreases, in other words, the animal must forage for longer to maximize *R* when quality is lower. As such, previous claims that optimal foraging time and patch quality are positively associated (e.g. [1,22]) are not always correct; in fact, this positive trend does not hold in the standard marginal value theorem [24].

In our analysis, we have mentioned that *g*(*t*)/*t* can act as an indicator of foraging conditions. This measure is equivalent to the prey encounter event (PEE) rate used as a proxy for prey encounter density [39] and is similar to the catch per unit effort (CPUE) as applied to diving birds [40,41]. In our notation, CPUE = *g*(*t*)/*u*, is the rate of gain while diving, whereas *g*(*t*)/*t* is the rate of gain while foraging. As previously discussed, this measure could be established from various methods such as direct measurements of prey capture with on-board cameras to more indirect measures using prey load on the last diving bout. It is, however, important to note that *g*(*t*)/*t*, while being an indicator of foraging conditions, is not equivalent to *x*. The former's dimensions are energy over time, it depends on *A* and can vary over the foraging duration of the dive, while the latter is dimensionless, independent of *A* and constant during the dive.

Correlations between the IPQ and other metrics have so far been conducted in six species (four alcid seabirds and two species of seal), with a few studies directly measuring prey abundance or value (table 1). Three of these species, Brünnich's, common, and black guillemots, bring a single-prey item to the nest [30], although within their last dive, they may eat smaller items and return with the first item above a certain size (see [42] for an analysis). Given that all gain functions are based on time allocation models which are appropriate for multiple-prey loaders but less so for single-prey loaders [9], correlations with characteristics of the single prey brought to the nest cannot be taken as robust support for the use of the IPQ in those species. Correlations in the seal species provide stronger support. Yet, without a theoretical basis, correlations in a few cases do not provide convincing justification for a general use of the IPQ as a measure to estimate prey abundance or value at a foraging patch from dive data.

## Data accessibility

This article has no additional data.
